# Supplementary material for: Leveraging biogenic resources to achieve global plastic decarbonization by 2050
Source: Nat Commun. 2025 Aug 18;16:7659. doi: 10.1038/s41467-025-62877-6 (PMC12361497; doi:10.1038/s41467-025-62877-6)
Supplement: Supplementary file 1 — Supplementary Information [file 41467_2025_62877_MOESM1_ESM.pdf]

Supplementary Information for

Leveraging biogenic resources to achieve global plastic decarbonization by 2050

Elisabeth Van Roijen [1, *], Sabbie A. Miller [1]
[1] Department of Civil and Environmental Engineering, University of California, Davis, United States of America
[*] Corresponding Author: E evanroijen@ucdavis.edu

This PDF file includes:

Supplementary Methods

Other supporting materials for this manuscript include the following:

Supplementary dataset S1 to S13

Supplementary methods

Supplementary methods 1. Cradle-to-gate plastic impacts. The cradle-to-gate impacts (namely the combined GWP of CO_2, CH_4 and N_2O emissions) for bio-based plastic production were derived from [1] using a mass-based allocation approach. When multiple bio-based feedstocks were presented, an average value was used. For example, Van Roijen and Miller [1] present results for bio-based PLA made from both corn stover and sugarcane bagasse. Therefore, in this study, the GHG emissions of both production routes are averaged together to obtain an estimate for the cradle-to-gate impacts of PLA from 2nd generation feedstocks. See supplemental dataset, Sheet 3, for a full list of these assumptions. The LCI for the 2018 global average electricity grid, as well as the renewable energy scenario (wind electricity, and biogas energy), come from [1]. The impacts for petroleum-based plastic were derived from ecoinvent [2]. The only modification made to the petroleum-plastic inventories was the electricity grid, which was either set to the 2018 global average electricity grid or wind electricity depending on the scenario.

Supplementary methods 2. End-of-life plastic impacts. The GHG emissions associated with bio-based plastic disposal in landfills, incineration, composting, anaerobic digestion, and mechanical recycling come from [3]. The impacts of chemical recycling of mixed plastic waste via pyrolysis was calculated using the inventory reported in [4] and by applying CO_2, CH_4 and N_2O GWP factors reported in the sixth IPCC report [5]. The only modification made to the inventory was the electricity grid, which was either swapped out with the 2018 global average, or wind electricity depending on the scenario. A similar approach was utilized to calculate the impacts of alternative chemical recycling methods (e.g. glycolysis, hydrolysis) reported in literature (See Supplemental Dataset, Sheet 7).

Supplementary methods 3. Identifying net-negative GHG scenarios. The weighted average production and end-of-life emissions were combined to determine the total impacts per kg of plastic under various scenarios. The emissions associated with plastic production were calculated by taking a weighted average of the carbon footprints of plastics based on their projected market share. Similarly, a weighted average of end-of-life emissions were determined assuming the make-up of plastics at end-of-life is the same as the make-up of plastic production. Scenarios resulting in a GWP of less than -0.1 kg CO_2e/kg plastic were considered net-negative GHG scenarios and are reported in Supplemental Dataset, Sheet 8. Aside from the total emissions (which are reported in terms of kg CO_2e/kg plastic), all other variables are reported as a decimal from 0 to 1, representing the fraction of implementation. For example, a chemical recycling value of 0.2 for biodegradable plastics signifies that 20% of biodegradable bio-based plastics are disposed of via chemical recycling (specifically pyrolysis). Similarly, a value of 0.9 for "bio-based plastic market", means that 90% (by weight) of plastics are assumed to be made using bio-based resources. It is important to note that only 2nd/3rd generation feedstocks were considered as bio-based resources when examining net-negative GHG emission to avoid competition with food.

Supplementary methods 4. Resource availability. The global annual production of feedstocks was derived from FAO [6]. The availability of used cooking oil was determined by taking the total annual production of vegetable oil, and multiplying it by the expected yield of used cooking oil reported in literature [7]. The availability of biomethane was determined using global biomethane potential (reported in MJ) [8] and the energy density of biomethane [9] . Availability of wheat straw and corn stover were determined assuming that 30% and 2/3 of the residues are needed on the field as a soil amendment, as reported in [1]. Furthermore, the ratio of byproduct yield to main crop for corn and wheat were assumed to be 1 and 1.3, respectively [1]. The annual production of sugarcane molasses was calculated assuming a 5% weight yield of molasses per kg of sugarcane production [1].

Supplementary methods 5. Roadmap to 2050. The global annual emissions for plastics were determined by combining the weighted-average production and end-of-life impacts per kg of

plastic, and scaling the emissions based on plastic demand. It was assumed that the make-up of the plastic market will stay the same in the future (with the exception of bio-based plastic alternatives being substituted in). The market make-up of plastics was therefore determined using the 2022 market distribution of plastic [10] as a baseline, and then determining bio-based plastic market replacements based on their technical substitution potential as reported in supplemental data sheet 1 (SDS1). These emissions were then scaled globally by multiplying the calculated carbon intensity by the anticipated mass of plastic consumption in that year. To account for the impact of plastic recycling on annual plastic production, the total mass of recycled plastic is multiplied by a substitution factor of 0.9 or 1 for thermomechanical and chemical recycling respectively [11], and then subtracted from the total demand to determine the quantity of virgin plastic production. The annual consumption of plastics was determined assuming an annual growth in plastic demand of 4% per year. From there, the quantity of virgin plastic production was calculated by subtracting the quantity of plastics that were anticipated to be recycled in that year.

Supplementary References

[1] E. C. Van Roijen and S. A. Miller, "Towards the production of net-negative greenhouse gas emission bio-based plastics from 2nd and 3rd generation feedstocks," *J. Clean. Prod.*, vol. 445, no. August 2023, p. 141203, 2024, doi: 10.1016/j.jclepro.2024.141203.

[2] R. Frischknecht *et al.*, "The ecoinvent database: Overview and methodological framework." International Journal of Life Cycle Assessment, 2005.

[3] E. C. Van Roijen and S. A. Miller, "A review of bioplastics at end-of-life : Linking experimental biodegradation studies and life cycle impact assessments," *Resour. Conserv. Recycl.*, vol. 181, no. January, p. 106236, 2022, doi: 10.1016/j.resconrec.2022.106236.

[4] H. Jeswani *et al.*, "Life cycle environmental impacts of chemical recycling via pyrolysis of mixed plastic waste in comparison with mechanical recycling and energy recovery," *Sci. Total Environ.*, vol. 769, 2021, doi: 10.1016/j.scitotenv.2020.144483.

[5] United Nations Environment programme, "Climate Change 2023: Synthesis Report. Contribution of Working Groups I, II and III to the Sixth Assessment Report of the Intergovernmental Panel on Climate Change," pp. 35–115, 2023, doi: 10.59327/IPCC/AR6-9789291691647.

[6] Food and Agriculture Organization of the United Nations, "Food and Agriculture Data." https://www.fao.org/faostat/en/#home.

[7] M. R. Teixeira, R. Nogueira, and L. M. Nunes, "Quantitative assessment of the valorisation of used cooking oils in 23 countries," *Waste Manag.*, vol. 78, pp. 611–620, 2018, doi: 10.1016/j.wasman.2018.06.039.

[8] J. Koornneef, P. Van Breevoort, P. Noothout, C. Hendriks, L. Luning, and A. Camps, "Global potential for biomethane production with carbon capture, transport and storage up to 2050," *Energy Procedia*, vol. 37, pp. 6043–6052, 2013, doi: 10.1016/j.egypro.2013.06.533.

[9] IEA, "An introduction to biogas and biomethane." https://www.iea.org/reports/outlook-for-biogas-and-biomethane-prospects-for-organic-growth/an-introduction-to-biogas-and-biomethane (accessed Aug. 04, 2024).

[10] Plastics Europe, "Plastics – the Facts 2022," 2022. [Online]. Available: https://plasticseurope.org/knowledge-hub/plastics-the-facts-2022/.

[11] P. Stegmann, V. Daioglou, M. Londo, and D. P. Van Vuuren, "Plastic futures and their CO 2 emissions," vol. 612, no. December, 2022, doi: 10.1038/s41586-022-05422-5.
