# Supplementary material for: Leveraging biogenic resources to achieve global plastic decarbonization by 2050
Source: Nat Commun. 2025 Aug 18;16:7659. doi: 10.1038/s41467-025-62877-6 (PMC12361497; doi:10.1038/s41467-025-62877-6)
Supplement: Supplementary file 2 — Description of Additional Supplementary File [file 41467_2025_62877_MOESM2_ESM.pdf]

Description of Additional Supplementary File

Supplementary Data 1 - Dataset containing LCI’s, assumptions, source data, and detailed results.
